# Strontium hexa­fluorido­stannate(IV) dihydrate

**DOI:** 10.1107/S2414314626007868

**Published:** 2026-08-04

**Authors:** Hans Reuter

**Affiliations:** a Chemistry, Osnabrück University, Barabarstr. 7, 49069 Osnabrück, Germany; Vienna University of Technology, Austria

**Keywords:** crystal structure, hexa­fluorido­stannate(IV), coordination polyhedra, hydrogen bonds, isotypism

## Abstract

Colourless, needle-like single crystals of the title compound, Sr[SnF_6_]·2H_2_O, were grown from stoichiometric amounts of SrF_2_ and SnF_4_ in boiling water. The compound is isostructural with the corresponding hexa­fluorido­iridate(IV) and features a nearly undistorted octa­hedral [SnF_6_]^2–^ anion with *C*_1_ point-group symmetry, as well as an Sr^2+^ ion in a distorted, square-anti­prismatic coordination environment. The resulting [SrF_5_(H_2_O)_3_] coordination polyhedra share an edge *via* two water mol­ecules to form centrosymmetric dimers. As the strength of the O—H⋯F and O—H⋯O hydrogen bonds is less pronounced, the crystal packing is dominated by *μ*_2_-F atoms between anions and cations.

## Abstract 2

Sr[SnF_6_]·2H_2_O is isostructural with the analogous hexa­fluorido­iridate(IV) and consists of almost undistorted [SnF_6_]^2–^ octa­hedra and of distorted [SrF_5_(H_2_O)_3_] square anti­prisms linked *via* common water mol­ecules into centrosymmetric dimers.

## Graphical abstract

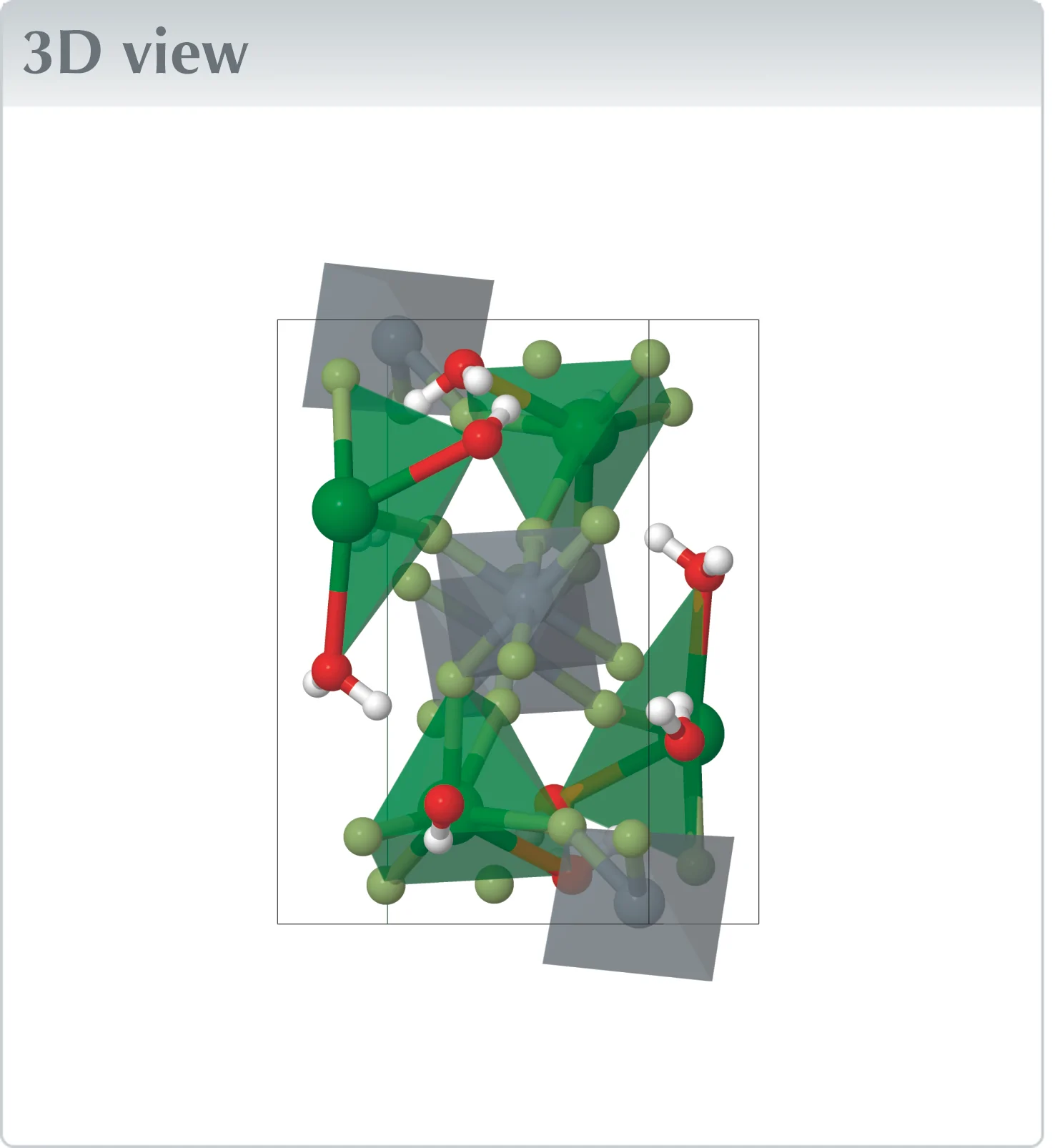

## Structure description

Hexa­fluorido­stannates(IV), [SnF_6_]^2–^, with monovalent and divalent metal cations have long been known (Marignac, 1859 ▸). They occur in both hydrated and anhydrous forms. Although they have frequently been studied crystallographically in the past (*e.g.* Hoppe *et al.*, 1972 ▸; Lari Lavassani *et al.*, 1974 ▸), complete, high-resolution single-crystal data sets based on diffractometer data are rare. Fe[SnF_6_]·6H_2_O belongs to this group (Denes *et al.*, 1994 ▸), but as its atomic coordinates have not been published or deposited, it cannot be found in any of the crystallographic databases. The situation is somewhat better in the case of simple ammonium and phospho­nium cations like (Me_3_NH)^+^ (dihydrate; Taha *et al.*, 1992 ▸) or (Ph_4_P)^+^ (anhydrous; Cortijo *et al.*, 2018 ▸). Today, the [SnF_6_]^2–^ anion is becoming increasingly important as a building block in coordination polymers (Li *et al.*, 2024 ▸; Wang *et al.*, 2024 ▸; Xiong *et al.*, 2024 ▸).

**Figure 1:**
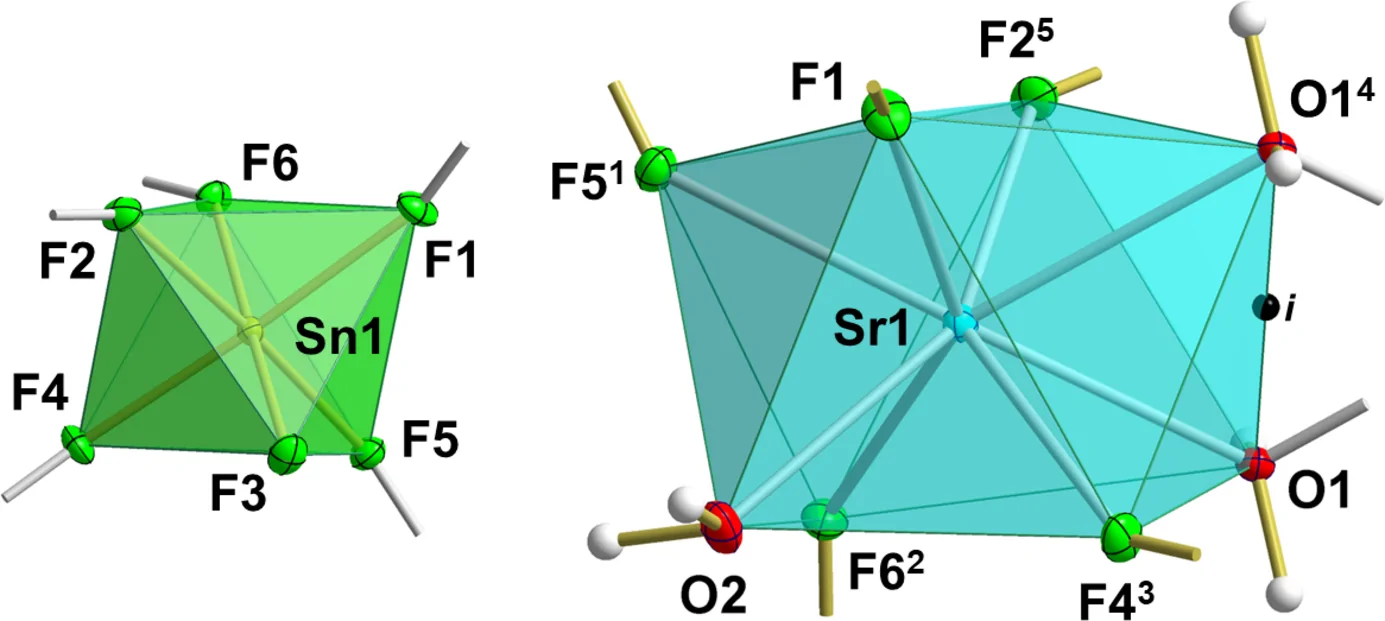
Coordination polyhedra around the metal atoms in Sr[SnF_6_]·2H_2_O with atom numbering. The almost octa­hedral [SnF_6_]^2–^ anion (green, left) and the distorted square-anti­prismatic [SrF_5_(H_2_O)_3_] polyhedron (blue, right) are on the same scale with all non-H atoms drawn as displacement ellipsoids at the 50% probability level. Covalent bonds are drawn in bronze, coordinative bonds are drawn in grey; the black dot labelled *i* indicates the position of the centre of symmetry generating the dimers of the strontium coordination polyhedra. [Symmetry codes: (1) 1 − *x*,1 − *y*,1 − *z*; (2) 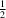 + *x*, 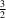 − *y*, −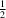 + *z*; (3) 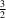 − *x*, 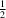 + *y*, 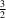 − *z*; (4) 1 − *x*, 2 − *y*, 1 − *z*; (5) −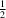 + *x*, 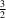 − *y*, −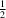 + *z*.]

The title compound, strontium hexa­fluorido­stannate(IV) dihydrate, is isostructural with the corresponding hexa­fluorido­iridate(IV) of strontium (Smolentsev *et al.*, 2007 ▸). In the [SnF_6_]^2–^ anion, point-group symmetry *C*_1_, Fig. 1 ▸, the tin–fluorine distances vary between 1.9356 (14) and 1.9573 (13) Å, mean value = 1.947 (10) Å, whilst the bond angles between the *trans* oriented fluorine atoms are 176.38 (6), 176.81 (6), and 177.37 (6)°. In other cases, these bond angles are exactly 180°, as the anion exhibits higher symmetry, and the Sn—F bond lengths are somewhat shorter as reported in older, room-temperature single-crystal studies with somewhat lower precision: Li_2_[SnF_6_]·2H_2_O, *d*(Sn—F) = 1.962–1.983 Å, mean value = 1.969 (11) Å, point-group symmetry *C*_2*h*_ (Marseglia & Brown, 1973 ▸); Na_2_[SnF_6_], *d*(Sn—F) = 6 × 1.959 Å, point-group symmetry *D*_2*h*_ (Benner & Hoppe, 1990 ▸); (Me_3_NH)_2_[SnF_6_]·2H_2_O, *d*(Sn—F) = 1.932 (9)–1.965 (3) Å, mean value = 1.965 (19) Å, point group symmetry *C*_2*h*_ (Taha *et al.*, 1992 ▸). In the tetra­phenyl­phospho­nium salt (Ph_4_P)_2_[SnF_6_], the anion also exhibits point-group symmetry *C*_1_, with Sn—F bond lengths between 1.953 (1) and 1.985 (1) Å, mean value 1.963 (14) Å, and *trans* bond angles of 179.01 (3)–178.59 (3)° (Cortijo *et al.*, 2018 ▸).

**Figure 2:**
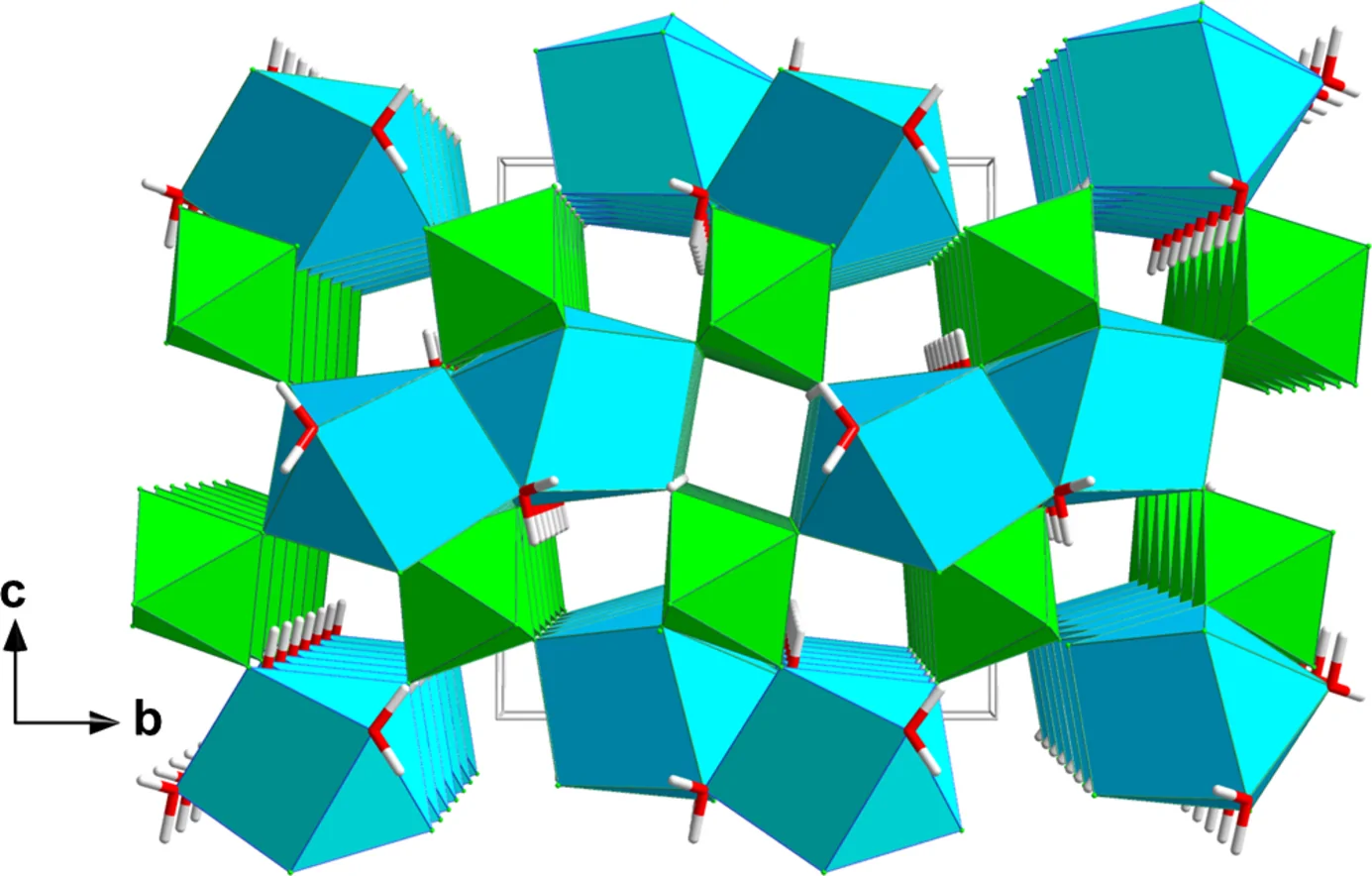
Polyhedral representation of the crystal packing looking down the *a* axis. [SnF_6_]^2−^ octa­hedra are drawn in green and the distorted [SrF_5_(H_2_O)_3_] square anti­prisms in blue; water mol­ecules are visualized as stick models.

Of the six fluorine atoms, all but one (F3) also coordinate to the strontium atom. This metal atom is surrounded by five fluorine atoms and three water mol­ecules, arranged in a distorted square-anti­prismatic configuration (Fig. 1 ▸). The Sr—F bond lengths range from 2.4252 (14)–2.4951 (14) Å, with a mean value of 2.453 (26) Å; the strontium–oxygen bond lengths are all slightly longer, ranging from 2.6183 (17)–2.7240 (16) Å. These values can be directly compared with those of the isostructural iridium compound [ambient temperature, *d*(Sr—F) = 2.421 (3)–2.515 (3) Å, mean value = 2.461 (34) Å, *d*(Sr—O) = 2.610 (4)–2.704 (4) Å]. Three water mol­ecules coordinate to the strontium atom, yet only two water mol­ecules appear in the mol­ecular formula, which is due to the fact that one water mol­ecule (O1) bridges two strontium atoms, resulting in dimers formed by edge-sharing square anti­prisms [Sr(μ_2_-F)_5_(μ_1_-H_2_O)(μ_2_-H_2_O)]. Together with the octa­hedral [SnF(μ_2_-F)_5_] building blocks, the compact packing shown in Fig. 2 ▸ results.

Both water mol­ecules are engaged in hydrogen-bonding inter­actions (Table 1 ▸). Based on the relatively long donor⋯acceptor distances (in particular for the O⋯O and one of the O⋯F contacts) and the acute bond angles at the hydrogen atoms, the hydrogen-bonding inter­actions are considered as medium-strong to weak.

## Synthesis and crystallization

In a beaker, 0.30 g (1.56 mmol) of SnF_4_ and 0.20 g (1.56 mmol) of SrF_2_ were suspended in 100 ml of water, and the mixture was heated to boiling for 10 min. under stirring. The unreacted reactants were filtered off whilst hot; colorless needles of the title compound crystallized as the solution cooled and the solvent evaporated.

## Refinement

Crystal data, data collection and structure refinement details are summarized in Table 2 ▸. The four H atoms of the water mol­ecules were clearly identified in difference-Fourier syntheses. Their positions were modeled with a common O—H distance of 0.96 Å and a H—O—H bond angle of 105.0° before they were fixed and allowed to ride on the corresponding oxygen atom with one common isotropic temperature factor.

## Supplementary Material

Crystal structure: contains datablock(s) I. DOI: 10.1107/S2414314626007868/wm4256sup1.cif

Structure factors: contains datablock(s) I. DOI: 10.1107/S2414314626007868/wm4256Isup2.hkl

CCDC reference: 2577756

Additional supporting information:  crystallographic information; 3D view; checkCIF report

## Figures and Tables

**Table 1:** Hydrogen-bond geometry (Å, °)

*D*—H⋯*A*	*D*—H	H⋯*A*	*D*⋯*A*	*D*—H⋯*A*
O1—H11⋯F3^i^	0.96	1.92	2.796 (2)	150
O1—H12⋯O2^ii^	0.96	2.06	2.986 (2)	161
O2—H21⋯F6^iii^	0.96	2.50	3.263 (2)	137
O2—H22⋯F3^iv^	0.96	1.81	2.764 (2)	173

Symmetry codes: (i) 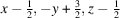; (ii) 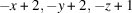; (iii) 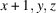; (iv) 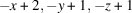.

**Table 2:** Experimental details

Crystal data	
Chemical formula	Sr[SnF_6_]·2H_2_O
*M*_r_	356.34
Crystal system, space group	Monoclinic, *P*2_1_/*n*
Temperature (K)	100
*a*, *b*, *c* (Å)	6.0782 (4), 9.8884 (6), 11.2968 (8)
β (°)	99.154 (3)
*V* (Å^3^)	670.33 (8)
*Z*	4
Radiation type	Mo *K*α
μ (mm^−1^)	11.75
Crystal size (mm)	0.29 × 0.07 × 0.07

Data collection	
Diffractometer	Bruker APEXII CCD
Absorption correction	Multi-scan (*SADABS*; Krause *et al.*, 2015 ▸)
*T*_min_, *T*_max_	0.458, 0.746
No. of measured, independent and observed [*I* > 2σ(*I*)] reflections	73429, 1947, 1842
*R*_int_	0.056
(sin θ/λ)_max_ (Å^−1^)	0.703

Refinement	
*R*[*F*^2^ > 2σ(*F*^2^)], *wR*(*F*^2^), *S*	0.014, 0.033, 1.09
No. of reflections	1947
No. of parameters	92
H-atom treatment	H-atom parameters constrained
Δρ_max_, Δρ_min_ (e Å^−3^)	0.45, −0.49

Computer programs: *APEX2* and *SAINT* (Bruker, 2009 ▸), *SHELXS7* (Sheldrick, 2008 ▸), *SHELXL7* (Sheldrick, 2015 ▸), *DIAMOND* (Brandenburg, 2006 ▸), *Mercury* (Macrae *et al.*, 2020 ▸) and *publCIF* (Westrip, 2010 ▸).

## full crystallographic data

### Strontium hexafluoridostannate(IV) dihydrate Crystal data

**Table:**

Sr[SnF_6_]·2H_2_O	*F*(000) = 648
*M**_r_* = 356.34	*D*_x_ = 3.531 Mg m^−^^3^
Monoclinic, *P*2_1_/*n*	Mo *K*α radiation, λ = 0.71073 Å
*a* = 6.0782 (4) Å	Cell parameters from 9976 reflections
*b* = 9.8884 (6) Å	θ = 2.8–30.0°
*c* = 11.2968 (8) Å	µ = 11.75 mm^−^^1^
β = 99.154 (3)°	*T* = 100 K
*V* = 670.33 (8) Å^3^	Prism, colourless
*Z* = 4	0.29 × 0.07 × 0.07 mm

### Strontium hexafluoridostannate(IV) dihydrate Data collection

**Table:**

Bruker APEXII CCD diffractometer	1842 reflections with *I* > 2σ(*I*)
φ and ω scans	*R*_int_ = 0.056
Absorption correction: multi-scan (SADABS; Krause *et al*., 2015)	θ_max_ = 30.0°, θ_min_ = 2.8°
*T*_min_ = 0.458, *T*_max_ = 0.746	*h* = −8→8
73429 measured reflections	*k* = −13→13
1947 independent reflections	*l* = −15→15

### Strontium hexafluoridostannate(IV) dihydrate Refinement

**Table:**

Refinement on *F*^2^	Primary atom site location: structure-invariant direct methods
Least-squares matrix: full	Hydrogen site location: difference Fourier map
*R*[*F*^2^ > 2σ(*F*^2^)] = 0.014	H-atom parameters constrained
*wR*(*F*^2^) = 0.033	*w* = 1/[σ^2^(*F*_o_^2^) + (0.0112*P*)^2^ + 1.2564*P*] where *P* = (*F*_o_^2^ + 2*F*_c_^2^)/3
*S* = 1.09	(Δ/σ)_max_ = 0.001
1947 reflections	Δρ_max_ = 0.45 e Å^−^^3^
92 parameters	Δρ_min_ = −0.49 e Å^−^^3^
0 restraints	

### Strontium hexafluoridostannate(IV) dihydrate Special details

**Table:**

Geometry. All esds (except the esd in the dihedral angle between two l.s. planes) are estimated using the full covariance matrix. The cell esds are taken into account individually in the estimation of esds in distances, angles and torsion angles; correlations between esds in cell parameters are only used when they are defined by crystal symmetry. An approximate (isotropic) treatment of cell esds is used for estimating esds involving l.s. planes.

### Strontium hexafluoridostannate(IV) dihydrate Fractional atomic coordinates and isotropic or equivalent isotropic displacement parameters (Å^2^)

**Table:**

	*x*	*y*	*z*	*U*_iso_*/*U*_eq_	
Sn1	0.59421 (2)	0.53509 (2)	0.73192 (2)	0.00724 (4)	
Sr1	0.66772 (3)	0.81470 (2)	0.45187 (2)	0.00759 (5)	
F1	0.5712 (2)	0.64125 (15)	0.58646 (13)	0.0163 (3)	
F2	0.8141 (2)	0.66012 (14)	0.81610 (13)	0.0157 (3)	
F3	0.8428 (2)	0.43410 (15)	0.68662 (13)	0.0166 (3)	
F4	0.6090 (2)	0.43590 (14)	0.88292 (12)	0.0151 (3)	
F5	0.3769 (2)	0.40503 (14)	0.65792 (12)	0.0146 (3)	
F6	0.3519 (2)	0.64425 (14)	0.77656 (12)	0.0140 (3)	
O1	0.6187 (3)	1.08124 (16)	0.40924 (14)	0.0104 (3)	
H11	0.5568	1.1011	0.3275	0.041 (5)*	
H12	0.7467	1.1390	0.4269	0.041 (5)*	
O2	1.0554 (3)	0.69766 (18)	0.51358 (16)	0.0169 (3)	
H21	1.0914	0.6397	0.5821	0.041 (5)*	
H22	1.1007	0.6482	0.4484	0.041 (5)*	

### Strontium hexafluoridostannate(IV) dihydrate Atomic displacement parameters (Å^2^)

**Table:**

	*U*^11^	*U*^22^	*U*^33^	*U*^12^	*U*^13^	*U*^23^
Sn1	0.00757 (7)	0.00717 (7)	0.00704 (7)	0.00000 (5)	0.00136 (5)	0.00017 (5)
Sr1	0.00768 (9)	0.00759 (9)	0.00753 (9)	0.00034 (7)	0.00130 (7)	−0.00075 (7)
F1	0.0195 (7)	0.0151 (7)	0.0160 (7)	0.0028 (6)	0.0076 (5)	0.0062 (5)
F2	0.0121 (6)	0.0125 (7)	0.0208 (7)	−0.0016 (5)	−0.0023 (5)	−0.0026 (5)
F3	0.0134 (6)	0.0191 (7)	0.0173 (7)	0.0045 (5)	0.0030 (5)	−0.0043 (6)
F4	0.0151 (7)	0.0173 (7)	0.0121 (6)	−0.0004 (5)	−0.0005 (5)	0.0065 (5)
F5	0.0149 (7)	0.0125 (6)	0.0155 (7)	−0.0005 (5)	0.0000 (5)	−0.0034 (5)
F6	0.0150 (7)	0.0143 (7)	0.0139 (7)	0.0018 (5)	0.0063 (5)	−0.0012 (5)
O1	0.0086 (7)	0.0104 (7)	0.0120 (7)	−0.0006 (6)	0.0010 (6)	−0.0007 (6)
O2	0.0131 (8)	0.0187 (9)	0.0181 (9)	0.0054 (7)	0.0003 (6)	−0.0027 (7)

### Strontium hexafluoridostannate(IV) dihydrate Geometric parameters (Å, º)

**Table:**

Sn1—F5	1.9356 (14)	Sr1—O1	2.6874 (16)
Sn1—F1	1.9361 (14)	Sr1—O1^v^	2.7240 (16)
Sn1—F3	1.9464 (14)	Sr1—Sr1^v^	4.4130 (4)
Sn1—F2	1.9530 (14)	F2—Sr1^vi^	2.4467 (14)
Sn1—F6	1.9558 (14)	F4—Sr1^vii^	2.4405 (13)
Sn1—F4	1.9573 (13)	F5—Sr1^iv^	2.4951 (14)
Sr1—F1	2.4252 (14)	F6—Sr1^viii^	2.4587 (13)
Sr1—F4^i^	2.4405 (14)	O1—Sr1^v^	2.7240 (16)
Sr1—F2^ii^	2.4467 (14)	O1—H11	0.9600
Sr1—F6^iii^	2.4587 (13)	O1—H12	0.9600
Sr1—F5^iv^	2.4951 (14)	O2—H21	0.9600
Sr1—O2	2.6183 (17)	O2—H22	0.9600
			
F5—Sn1—F1	92.38 (6)	F6^iii^—Sr1—O1	75.26 (5)
F5—Sn1—F3	92.90 (6)	F5^iv^—Sr1—O1	139.89 (5)
F1—Sn1—F3	90.54 (6)	O2—Sr1—O1	123.41 (5)
F5—Sn1—F2	176.38 (6)	F1—Sr1—O1^v^	70.46 (5)
F1—Sn1—F2	91.23 (6)	F4^i^—Sr1—O1^v^	72.70 (5)
F3—Sn1—F2	87.32 (6)	F2^ii^—Sr1—O1^v^	75.19 (5)
F5—Sn1—F6	89.30 (6)	F6^iii^—Sr1—O1^v^	144.70 (5)
F1—Sn1—F6	87.93 (6)	F5^iv^—Sr1—O1^v^	125.90 (5)
F3—Sn1—F6	177.37 (6)	O2—Sr1—O1^v^	130.10 (5)
F2—Sn1—F6	90.58 (6)	O1—Sr1—O1^v^	70.73 (6)
F5—Sn1—F4	88.68 (6)	F1—Sr1—Sr1^v^	105.39 (3)
F1—Sn1—F4	176.81 (6)	F4^i^—Sr1—Sr1^v^	68.05 (3)
F3—Sn1—F4	92.41 (6)	F2^ii^—Sr1—Sr1^v^	71.08 (3)
F2—Sn1—F4	87.70 (6)	F6^iii^—Sr1—Sr1^v^	110.41 (3)
F6—Sn1—F4	89.08 (6)	F5^iv^—Sr1—Sr1^v^	145.86 (3)
F1—Sr1—F4^i^	91.63 (5)	O2—Sr1—Sr1^v^	137.13 (4)
F1—Sr1—F2^ii^	100.89 (5)	O1—Sr1—Sr1^v^	35.64 (3)
F4^i^—Sr1—F2^ii^	139.09 (5)	O1^v^—Sr1—Sr1^v^	35.09 (3)
F1—Sr1—F6^iii^	143.93 (5)	Sn1—F1—Sr1	157.45 (8)
F4^i^—Sr1—F6^iii^	105.42 (5)	Sn1—F2—Sr1^vi^	146.56 (7)
F2^ii^—Sr1—F6^iii^	86.87 (5)	Sn1—F4—Sr1^vii^	148.80 (7)
F1—Sr1—F5^iv^	71.21 (5)	Sn1—F5—Sr1^iv^	143.71 (7)
F4^i^—Sr1—F5^iv^	144.19 (5)	Sn1—F6—Sr1^viii^	139.19 (7)
F2^ii^—Sr1—F5^iv^	76.22 (5)	Sr1—O1—Sr1^v^	109.28 (6)
F6^iii^—Sr1—F5^iv^	76.75 (5)	Sr1—O1—H11	112.9
F1—Sr1—O2	79.50 (5)	Sr1^v^—O1—H11	106.7
F4^i^—Sr1—O2	69.27 (5)	Sr1—O1—H12	118.9
F2^ii^—Sr1—O2	151.12 (5)	Sr1^v^—O1—H12	103.1
F6^iii^—Sr1—O2	77.39 (5)	H11—O1—H12	105.0
F5^iv^—Sr1—O2	76.67 (5)	Sr1—O2—H21	124.0
F1—Sr1—O1	140.80 (5)	Sr1—O2—H22	112.1
F4^i^—Sr1—O1	71.80 (5)	H21—O2—H22	105.0
F2^ii^—Sr1—O1	74.13 (5)		

Symmetry codes: (i) −*x*+3/2, *y*+1/2, −*z*+3/2; (ii) *x*−1/2, −*y*+3/2, *z*−1/2; (iii) *x*+1/2, −*y*+3/2, *z*−1/2; (iv) −*x*+1, −*y*+1, −*z*+1; (v) −*x*+1, −*y*+2, −*z*+1; (vi) *x*+1/2, −*y*+3/2, *z*+1/2; (vii) −*x*+3/2, *y*−1/2, −*z*+3/2; (viii) *x*−1/2, −*y*+3/2, *z*+1/2.

### Strontium hexafluoridostannate(IV) dihydrate Hydrogen-bond geometry (Å, º)

**Table:**

*D*—H···*A*	*D*—H	H···*A*	*D*···*A*	*D*—H···*A*
O1—H11···F3^ii^	0.96	1.92	2.796 (2)	150
O1—H12···O2^ix^	0.96	2.06	2.986 (2)	161
O2—H21···F6^x^	0.96	2.50	3.263 (2)	137
O2—H22···F3^xi^	0.96	1.81	2.764 (2)	173

Symmetry codes: (ii) *x*−1/2, −*y*+3/2, *z*−1/2; (ix) −*x*+2, −*y*+2, −*z*+1; (x) *x*+1, *y*, *z*; (xi) −*x*+2, −*y*+1, −*z*+1.
